# Study protocol of “CHAPS”: a randomized controlled trial protocol of Care Coordination for Health Promotion and Activities in Parkinson’s Disease to improve the quality of care for individuals with Parkinson’s disease

**DOI:** 10.1186/s12883-015-0506-y

**Published:** 2015-12-15

**Authors:** Karen Connor, Eric Cheng, Hilary C. Siebens, Martin L. Lee, Brian S. Mittman, David A. Ganz, Barbara Vickrey

**Affiliations:** PADRECC: Parkinson’s Disease Research, Education, and Clinical Center, VA Greater Los Angeles Healthcare System, 11301 Wilshire Blvd, B500, ML 127, Los Angeles, CA 90073 USA; Department of Neurology, Geffen School of Medicine, University of California Los Angeles, 710 Westwood Plaza, C109RNRC, Los Angeles, CA 90095 USA; Siebens Patient Care Communications, 13601 Del Monte Blvd, Suite 47A, Seal Beach, CA 90740 USA; Sepulveda VA Ambulatory Care Center, VA Greater Los Angeles Healthcare System, 16111 Plummer St., North Hills, CA 91343 USA; University of California Los Angeles Fielding School of Public Health, Department of Biostatistics, 405 Hilgard Ave, Los Angeles, CA 90024 USA; Center for Implementation Practice and Research Support (CIPRS), Veterans Affairs Greater Los Angeles Healthcare System (152), 16111 Plummer Street, Sepulveda, CA 91343 USA; Geriatric Research, Education and Clinical Center (GRECC), VA Greater Los Angeles Healthcare System, 11301 Wilshire Boulevard (11G), Building 158, Room 128, Los Angeles, CA 90073 USA

**Keywords:** Care coordination, Parkinson’s disease, Behavioral intervention, Clinical trial, Quality of health care, Nursing care management

## Abstract

**Background:**

Parkinson’s disease, the second most common neurodegenerative disease, is diagnostically defined by motor impairments, but also includes often under-recognized impairments in cognition, mood, sleep, and the autonomic nervous system. These problems can severely affect individuals’ quality of life. In our prior research, we have developed indicators to measure the quality of care delivered to patients with Parkinson’s disease, and we identified gaps in delivering evidence-based treatments for this population. Effective strategies to close these gaps are needed to improve patient quality of life.

**Methods/design:**

Building on prior research we developed a multi-faceted proactive implementation program called Care Coordination for Health Promotion and Activities in Parkinson’s Disease (CHAPS). To be eligible, patients had to have at least two visits with a primary diagnosis of idiopathic Parkinson’s disease (ICD-9 code: 332.0) at one of five Veterans Affairs Medical Centers in the southwestern United States from 2010 to 2014. The program consists of telephone assessments, evidence-based protocols, and tools to enhance patient self-management, care planning, and coordination of care across providers, including an electronic database to support and track coordination of care. Our mixed-methods study employs a randomized, controlled trial design to test whether the CHAPS intervention improves performance in 38 quality measures among an analytic sample of 346 patients. The 38 quality measures are categorized into overarching areas of communication, education, and continuity; regulatory reporting; diagnosis; periodic assessment; medication use; management of motor and non-motor symptoms; use of non-pharmacological approaches and therapies; palliative care; and health maintenance. Secondary outcomes are patient health-related quality of life, self-efficacy, and perceptions of care quality. We are also evaluating the extent of the CHAPS Program implementation and measuring program costs and impacts on health services utilization, in order to perform a analysis of the CHAPS program from the perspective of the Veterans Health Administration (VA). Outcomes are assessed by interviewer-administered surveys collected at baseline and at 6, 12, and 18 months, and by medical record chart abstractions. Analyses will be intention-to-treat.

**Discussion:**

The CHAPS Program is poised for dissemination within the VA National Parkinson’s Disease Research, Education, and Clinical Center Consortium if demonstrated efficacious.

**Trial Registration:**

ClinicalTrials.gov NCT01532986; registered on January 13, 2012.

## Background

Parkinson’s disease (PD) is the second most common neurodegenerative disease, after Alzheimer’s disease, in the United States (US) [1]. National estimates are that 60,000 new cases each year join approximately 1,500,000 individuals and their families that are afflicted with this disease. At onset, most are over age 65 and PD affects approximately 1.5 % of US residents 65 years and older. However, approximately 10–20 % of PD patients are under age 50, making this a disease that affects younger individuals as well [2].

Motor impairments of tremor, bradykinesia, postural instability, and rigidity define PD. Non-motor disturbances in cognition, mood, sleep, and the autonomic nervous system can also severely affect quality of life for persons with PD. While effective treatments exist, patients may not be receiving such care. We previously conducted a national survey that showed that primary care providers’ knowledge about PD care was much lower than that of general neurologists [3]. We developed quality of care indicators after performing a systematic review of the medical literature, then convening an expert panel to rate the indicators using a formal consensus method [4]. We subsequently conducted a structured medical chart review of 401 Veterans receiving care at the Veterans Affairs Greater Los Angeles Healthcare System to determine whether their care met the quality indicators that were judged as highest impact by the expert panel [5]. We determined that having been seen by movement disorder specialists was associated with higher quality of care than having been seen by general neurologists or by non-neurologists, particularly for treatment of advanced motor symptoms and assessment of non-motor symptoms. From this work, we inferred that a PD care intervention to promote best practices in PD care would require: 1) care protocols that include standardized assessment of PD motor and non-motor manifestations and 2) ongoing assessment and collaboration with, and referral to, subspecialists for management of certain motor manifestations or complications, as needed.

**Fig. 1:**
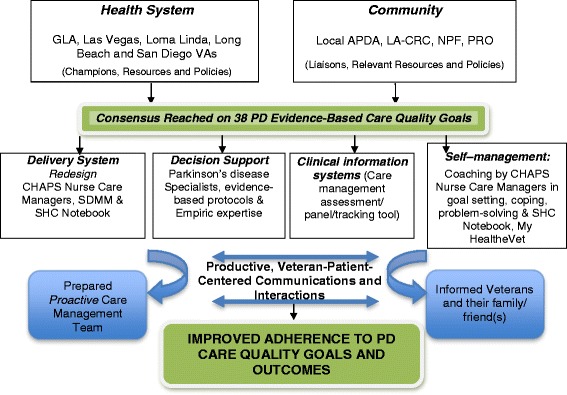
CHAPS Adaptation of the Chronic Care Model. Abbreviations: GLA—Greater Los Angeles; VAs—Veterans Health Administration; APDA—American Parkinson’s Disease Association; LA-CRC—Los Angeles Caregiver Resource Center; NPF—National Parkinson Foundation; PRO—Parkinson’s Resource Organization; CHAPS—Care Coordination for Health Promotion and Activities in Parkinson’s Disease; SDMM—Siebens Domain Management Model; SHC—Siebens Health Care

One approach to redesigning care to meet quality goals is the Chronic Care Model (CCM) (Fig. 1) [6–8]. The CCM identifies core system components to be addressed in chronic care redesign. Most CCM-based interventions have shown improvement of a care process or outcome measure and reduction of health-care costs as a result [7, 8]. Coordination of patient care is a key element addressed by the CCM. Studies addressing care coordination often show improved access to care and decreased hospitalizations [9, 10].

Care coordination, along with other CCM elements, was a critical component of our earlier work in dementia care and in the work of others [10–14]. In our Alzheimer’s Disease Coordinated Care for San Diego Seniors (ACCESS) study, a nurse- or social worker-led intervention included structured assessment, care protocols, collaborative care planning, coordination with care managers at community agencies, and proactive follow-up and tracking, utilizing a care management software program [11]. Among the positive outcomes were substantial improvement in the quality of care. Adherence to dementia care guidelines nearly doubled. Outcomes included better patient health-related quality of life, and fewer unmet caregiving assistance needs. Telephone or on-site home assessments were associated with better caregiver mastery [15]. Caregiver self-efficacy improved as well.

Improving patient and caregiver self-efficacy is an important goal in efforts to improve health care quality. The nursing profession’s biopsychosocial environmental construct ideally supports a self-efficacy focus in care coordination and management. Nurses bring attention to the strengths of individuals. This in turn helps to maximize self-management of PD and, ultimately, improvement in individuals’ quality of life [16]. Self-management tools tailored to PD aim to help Veterans identify health problems and apply solutions to reach specific goals, such as controlling symptoms and knowing how health problems affect quality of life [7, 8]. Areas of focus include integrating medication regimens into daily life, noting symptoms and treatment effects, and learning skills and strategies for successfully coping with difficult emotions. These approaches—the CCM Model and the self-efficacy concept—are applicable to improving PD care and can be integrated into care coordination programs.

This study’s primary specific aim is to test a nurse-led intervention/program, Care Coordination for Health Promotion and Activities in Parkinson’s Disease (CHAPS). We hypothesize that the CHAPS Program will increase adherence to evidence-based practice guidelines and improve outcomes of health-related quality of life and perceptions of care quality at a cost deemed feasible from the perspective of the Veterans Health Administration (VA).

## Methods/design

The intervention being assessed, a multi-component nurse-led care coordination clinical program, is based on adaptations of our earlier research. The overall research design is a multi-site, single-blinded, patient-level randomized-controlled trial of the intervention relative to usual care. Analyses include both primary and secondary outcomes as well as cost-related measures.

## Setting, population, and subjects for testing the intervention

The setting for this study includes five medical centers within the Veteran Integrated Service Network (VISN) 22 in the southwest United States. These medical centers are Greater Los Angeles, Las Vegas, Loma Linda, Long Beach, and San Diego. They are part of the Southwest Parkinson’s Disease Research, Education, and Clinical Centers (PADRECC), which is one of eight regional networks in the national VA PADRECC consortium. Institutional Review Board study approval was obtained at all sites.

Potential subjects are identified through a list of eligible Veterans with at least two International Classification of Diseases, Ninth Revision (ICD-9) diagnostic codes for PD (332.0) in administrative data through a query of the Data Warehouse for VISN 22 from October 1, 2010 through December 31, 2014 or until full enrollment is achieved [17].

Study *inclusion criteria* are: 1) Veterans with a diagnosis of Parkinson’s disease who 1) have had at least two visits between October 1, 2010 up to December 31, 2014 to one of the study sites; 2) are at least 18 years of age; and 3) demonstrate ability to provide consent for study participation. This is determined using screening questions (SAFE VET Mini Quiz) for “ability to participate in research” to identify if help is required in communicating with the research staff.

The *exclusion criteria* are: 1) Veteran is unable to participate in a dialog over the telephone, even with the help of his or her family or friend, 2) current enrollment in the Deep Brain Stimulation VA cooperative study, 3) enrollment in the Care Coordination Home Telehealth program; and 4) Veterans with PD and dementia who are unable to provide informed consent.

## Development of intervention

**Fig. 2:**
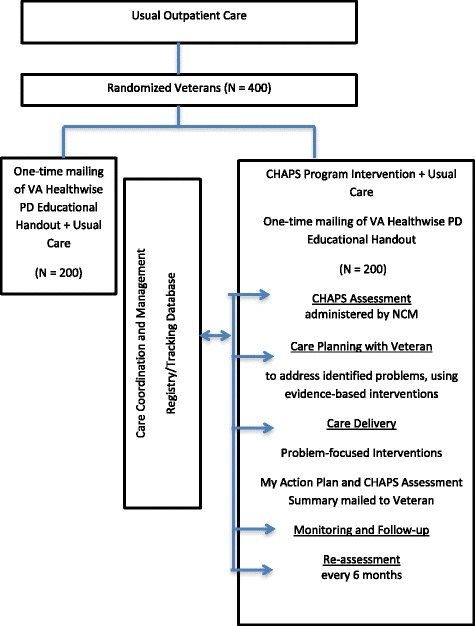
Study design and CHAPS Program intervention

Building on prior experiences in the ACCESS project and related published literature, we designed an outpatient PD care program addressing current limitations in PD care delivery based on the CCM (Fig. 1). In CHAPS, nurse care managers (NCMs) conduct structured assessments via telephone to proactively identify problems and unmet needs (Fig. 2). These trigger protocols for delivery of evidence-based, coordinated PD treatment guidelines in concert with Veterans’ priorities, VA providers’ expertise, and local community resources. NCMs make telephone calls to Veterans, send printed materials to instruct and support self-management, and conduct clinical huddles with neurologists, to coordinate PD care management proactively. This program overlays the existing care delivery structure of neurologist visit-based care. In addition, steps are embedded in the NCM protocols to address 38 PD quality indicators (Table 1). Through these approaches and procedures, the CHAPS Program is designed to proactively enhance and expand the ongoing PADRECC specialists’ care.

**Table 1:** Outcome measures—quality indicators for Parkinson’s disease care outcome measures

Communication, Education, and Continuity Indicators:
1. Because people with PD may develop impaired cognitive ability, a communication deficit and/or depression, they should be provided with: both oral and written communication throughout the course of the disease, which should be individually tailored and reinforced as necessary consistent communication from professionals involved
2. Families and caregivers should be given information about PD and PD with dementia (PDD) (if applicable), standards for diagnosis and symptom assessment, clinical and social services for which they are eligible, and the support services available including caregiver resources and dementia care (if applicable).
3. All Veterans with PD and their caregivers (if applicable) should be referred to one or more Parkinson’s disease advocacy and support organizations for information, education, and support including caregiver resources and dementia care (if applicable). (Referral to PD advocacy and support organizations)
4. People with PD and their caregivers should be given the opportunity to discuss end-of-life issues with appropriate healthcare professionals.
5. People with PD should have a comprehensive care plan agreed between the individual, their family and/or caregivers and specialist and secondary healthcare providers.
6. All Veterans with PD should be able to identify a provider or a clinic that they would call when in need of medical care or should know the phone number or other mechanism by which they can reach this source of care. (Identify source of care)
Reporting Indicators:
7. If a Veteran with PD or his or her family expresses concern about driving safely, then the clinician should advise the patient not to drive a motor vehicle and/or request the DMV retest the patients’ ability to drive, and/or refer the patient to a driver’s safety course that includes assessment of driving ability, in accordance with state laws. (Actions regarding driving safety concerns)
8. All Veterans with PD who report excessive daytime sleepiness should be instructed not to drive a motor vehicle. (Excessive daytime somnolence and driving restrictions)
9. All Veterans with PD who are wheelchair bound or demented should be assessed for evidence of abuse (physical, sexual, financial, neglect, isolation, abandonment). (Assessment for abuse)
Diagnosing Parkinson’s Disease:
10. The diagnosis of PD should be reviewed regularly (6–12 month intervals seen to review diagnosis) and re-considered if atypical clinical features develop.
Assessment:
11. All Veterans with PD, on at least an annual basis, should be assessed for the following:
Ability to operate a motor vehicle. (Assessment of driving ability in PD patients)
Depressive symptomotology
Dementia
Excessive daytime somnolence
Presence or absence of UI during the *initial evaluation and every 2 years thereafter.*
Functional status. (Assessment of functional status)
Speech and swallowing difficulties
Orthostatic hypotension
Gastro-intestinal symptoms including constipation
Psychosis, hallucinations and delirium
Erectile dysfunction
Weight *(at every visit)*
Occurrence of recent falls
Medication Use:
12. If a Veteran with PD is prescribed a new drug, then the prescribed drug should have a clearly defined indication documented in the medical record. (Documented indication for newly prescribed medication)
13. For all Veterans with PD, the outpatient medical record of every physician should contain an up-to-date medication list. (Up-to-date medication list)
14. If a Veteran with a new diagnosis of PD has impairment in activities of daily living and is prescribed either levodopa or a dopamine agonist (DA), then the tradeoffs of initiating dopamine agonists versus levodopa should be discussed with the patient. (Dopamine agonist vs. levodopa as initial treatment)
15. If a patient has PD and has motor fluctuations, and is prescribed levodopa, then he or she should be educated about timing of intake of dietary amino acids and its impact on response to levodopa. (Timing of levodopa and dietary amino acids)
16. Clinicians should be aware of dopamine dysregulation syndrome, an uncommon disorder in which dopaminergic medication misuse is associated with abnormal behaviors, including hypersexuality, pathological gambling and stereotypic motor acts. This syndrome may be difficult to manage.
Management of Motor Symptoms and Dystonias:
17. If a Veteran is receiving therapy with a dopaminergic agent (levodopa or a dopamine agonist), then they should be assessed for the presence of motor complications (wearing-off, on-off fluctuations, or dyskinesia) at least every 6 months. (Assessment of motor complications)
18. Off-Period and Early Morning Dystonias Usual strategies for wearing-off can be applied in cases of off-period dystonia.
Management of Non-Motor Complications of Parkinson’s Disease:
19. If a patient with PD is newly treated for depression, then degree of response to at least two of the nine DSM-IV target symptoms for major depression and, if he or she is taking antidepressant medications, medication side effects should be documented at the first follow-up visit to the same physician or to a mental health provider within 4 weeks of treatment initiation.

### Intervention staffing

The central feature of the CHAPS Program is delivery system re-design through creation of the proactive CHAPS nurse care manager role. This clinician leads the execution and coordination of all the care management activities to be carried out with Veterans. Nurses may have different backgrounds yet are required to have outpatient care experience. Because they are part of the clinical VA staff, they have full access to clinical resources and clinical documentation systems.

### Care management components

Each participating Veteran receives an initial telephone call from the NCM to introduce him or herself as the CHAPS Program nurse care manager, and to schedule a time to start the CHAPS Assessment (Fig. 2) with the Veteran. The NCM administers the comprehensive structured assessment via telephone and then follows up periodically. The assessment, programmed in Microsoft (MS) Access, is based on existing validated and standardized instruments (e.g., Patient Health Questionnaire (PHQ)-9, World Health Organization (WHO)-5, subscales of the Movement Disorder Society-United Parkinson’s Disease Revised Scale (MDS-UPDRS)) and other questions developed by the research team as currently in use in other health care settings (Table 2). Some of these items, like the Epworth Sleep Scale, have been adapted to fit this patient population [18]. Based on pilot work that identified unmet care needs by Veterans, assessment algorithms (triggers) are included in the assessment to identify 28 priority problem areas (Table 2). They are categorized as routine or urgent. For example, a score of 10–17 points on the Epworth Sleep Scale would be categorized as a routine problem, but a score 18 points or greater on this scale would be categorized as an urgent problem [19].

**Table 2:** Comprehensive CHAPS assessment items and priority problem areas and associated standard care plans

CHAPS assessment	Priority problems/standard care plans
I. Medical/Surgical Issues (The Body) ^a^	
Medication Reconciliation	1 Prevention
Motor Complications—Part IV of MDS-UPDRS (2008) [44]	2 Medication
	3 Motor-related
Non-motor Complications - adapted from PD-HRQoL [45], MDS-UPDRS [44]^,^ ADL-UPDRS [46]	4 Gastrointestinal-related
	5 Weight/Nutrition/Dental
PD Sleep Scale [18]	6 Swallowing
Daytime Sleepiness—Epworth Sleepiness Scale [19]	7 Urology-related
	8 Pain
	9 Sleep and Fatigue
II. Mental Status/Emotions/Coping (The Mind)	
Montreal Cognitive Assessment (MoCA)-BLIND [47]	10 Hearing
Depression Screening—WHO-5 (depression screening in PD) and PHQ-9 [48, 49]	11 Vision
	12 Speech
Health Literacy—2 questions adapted from L Chew [50]	13 Cognitive Impairment
Internet Use—www.pewinternet.org	14 Psychosis/Hallucinations
Apathy—MDS-UPDRS [44]	15 Depression
Preferences—Advance Directives—Adapted from ACCESS [11]	16 Anxiety
	17 Understanding Parkinson’s Disease
Long-term Planning—End of Life Resources—ACCESS [11]	
	18 Coping/Self-management
	19 Apathy
Perception of Health—Short Form-36 [51]	20 Impulse Control Disorder
	21 Preferences/Long term care planning
III. Physical Function (Activities)	
Functional Limitations—Adapted from PD-HRQoL [45]	22 Functional Limitations
Social Isolation—PDQ-39 (IADL) [52]	23 Falls
Sense of Social Support—ACCESS Caregiver Survey [11]	24 Physical Activity
	25 Driving
Falls—Adapted NMSS [53] and ACCESS Falls [11]	
Exercise—MOS Physical Activity Items [54]	
Driving Ability—Elderly Drivers Checklist	
http://www.aging-parents-and-elder-care.com/Pages/Checklists/Elderly_Drivers.html	
IV. Living Environment (Surroundings)	
Community Agency Awareness—Adapted from ACCESS [11]	26 Elder Abuse
	27 Access to Care
Access to Care—Adapted from MS Survey [53]	28 End of Life Resources
Elder Abuse Screen—HWALEK-SENGSTOCK Elder	
Abuse Screening Test (H-S.EAST) [55]	

^a^ Section headings from Siebens Domain Management Model © Hilary C Siebens MD 2005, used with permission

*MDS-UPDRS* Movement Disorders Society—Unified Parkinson’s Disease Rating Scale; *ADL-UPDRS* Activities of Daily Living-Unified Parkinson’s Disease Rating Scale; *WHO* World Health Organization; *PD* Parkinson’s disease; *PHQ* Patient Health Questionnaire; *ACCESS* Alzheimer’s Disease Coordinated Care for San Diego Seniors; *PD-HRQoL* Parkinson’s disease-Health-related quality of life; *PDQ* Parkinson’s Disease Questionnaire; *IADL* intermediate or instrumental activities of daily living; *NMSS* National Multiple Sclerosis Society; *MOS* Medical Outcomes Study. *MS* Multiple Sclerosis

### Care management

The NCM schedules follow-up calls, adjusted to meet the Veteran’s needs. The NCM follows a standardized process documented in a hard-copy Care Coordination Binder that is also available electronically. It includes care plans and national and regional resources for each of the 28 priority problems (Table 2). The care plans are structured with suggestions in each of five steps: 1) Assess Further, 2) Inform by Providing Materials and Education, 3) Problem-solving and Self-Management/Self-care, 4) Clinical Referral or Follow-Up, and 5) Resources in Community and VA Social Services.

Each site identifies a CHAPS Program neurologist to serves as a champion for the Program. NCMs communicate once a month, either, in-person, by telephone, or by encrypted email with this neurologist in a “clinical huddle”. Together the NCM and neurologist review specific problems and clarify care coordination needs. All care is documented in the VA’s electronic medical record for optimal communication with all care providers within the VA.

### Self-management

NCM coaching actions support self-management by including goal setting, and use of self-management tools such as “My Action Plan” and provision of relevant print and/or electronic educational material. Regularly scheduled NCM telephone team meetings, facilitated by a team leader, further support the self-management component to problem solve specific challenges.

### Materials for veterans

CHAPS NCMs utilize MS Word templates for the self-management care plan, My Action Plan. The focus of the initial Action Plan is orientation to the self-management healthcare notebook and a few priority problems that the Veteran and NCM collaboratively decide are the most important to work on. These Veteran-focused personalized action plans are used as reminders to the Veteran about changes in lifestyle habits and other suggestions that ideally will take place between encounters. The initial self-management care plans are printed and mailed to Veterans in a 3-ringed binder that is customized for each Veteran. A printed hard copy of the initial CHAPS Assessment is included as well. The notebook provides Veterans with a method for keeping their important health information in one place, for their own reference and/or for their family and/or friend(s) who assist the Veteran with day-to-day activities. This approach facilitates communication among patients’ multiple providers. Because many Veterans receive care outside the VA where their electronic medical information will not be available, a printed binder given to patients provides one option for improving communication, and in turn care coordination, among VA and non-VA providers. In addition, research on learning and reading suggests differences in the use of paper versus screen presentations [20, 21]. For example, students showed better reading comprehension of printed texts compared to digital texts. In addition, not all Veterans have access to or use the Internet. Therefore, distributing a judicious amount of print material in a self-management tool, like a 3-ringed binder, was felt to be essential.

Additional educational materials are mailed to the Veteran when required to work on new problems. An updated “My Action Plan” is mailed after the yearly reassessment, or more often if the nurse and Veteran decide that might be beneficial. CHAPS NCMs also urge Veterans to use My HealtheVet, an online personal health record that also offers secure messaging with the Veteran’s provider. As part of total care management, if a Veteran in the intervention group states that he/she has a family or friend caregiver during the administration of the CHAPS Assessment, the NCM sends the Veteran a *CHAPS Intervention Caregiver Support Packet* that contains caregiver assessment questions regarding depression, strain, social isolation, and overall health and caregiver resource organizations.

### Hospital admission notification process

The team established a “Hospital Admission Notification” in the VA’s computerized patient records system (CPRS), at each site, to alert the respective CHAPS NCM when an intervention patient is admitted to a VA hospital. This provides NCMs the opportunity to contact families to be proactive in hospital care, as it relates to PD, especially regarding PD medications. This notification facilitates post-hospital PD care delivery as well.

## Outcome measures

In 2011, we convened a Task Force from four regional VA PADRECC medical centers (Greater Los Angeles, Las Vegas, Loma Linda, and Long Beach) and included five local PD advocacy and service organizations. Through a formal consensus process, the Task Force selected 38 PD quality of care indicators as having the most room for improvement and being of high value for treatment and management of PD (Table 1). These indicators were drawn from a larger set of 106 indicators developed from national and international work [4, 22].

### Secondary outcomes

These are listed in Table 3 and will each be measured using standardized, established measures.

**Table 3:** Secondary outcome measures to evaluate CHAPS program

Patient health-related quality of life (Health Utilities Index) [56]	
Depression (WHO-5, PHQ-9) [48, 49]	
Patient Self-efficacy (General Self-efficacy Scale) [57]	
Patient perceptions of care quality (Consumer Assessment of Health Plan) [58] and Patient Assessment of Care for Chronic Conditions (PACIC) [59]	
Functional Status (Subscale of UPDRS-ADL subscale) [46]	
Social support (MOS Social Support Survey) [60]	
Comorbidity (Charlson Comorbitiy Index) [61]	
Health services utilization (VISN-22 Data Warehouse) [35]	

*WHO* World Health Organization; *PHQ* Patient Health Questionnaire; *UPDRS-ADL* Unified Parkinson’s Disease Rating Scale—Activities of Daily Living; *MOS* Medical Outcomes Study; *VISN* Veteran Integrated Service Network

### Intervention costs

(1) *Start-up costs* to implement the intervention, such as computer hardware, software development, and items required for care manager training, are collected. Start-up costs are also tracked using the Intervention Activities Log, which staff use to record time for one-time intervention activities. (2) *Maintenance costs* will be estimated by the Cost Assessment Activity logs, which collect the frequency and time required for on-going care management activities and resources required.

## Research design

The overall research design is a randomized controlled trial involving a comparison between arm group receiving the nurse-led CHAPS Program intervention and a control arm receiving care as usual (Fig. 2). Research staff assessors collecting outcome data are unaware of assignment arm so they are not influenced by that knowledge.

### Randomization

Before enrollment began, the programmer created randomization tables that assigned subjects in a 1:1 ratio to either the intervention arm or control arm. The tables had a block size of 4 and were stratified by site. After the research staff assessor administers the baseline interview to consented study participants, the program manager uses the randomization table to assign study arm. The program manager then notifies the nurse care managers of assigned intervention patients. To minimize potential bias from participants’ awareness of randomization arm assignment, all study participants (both intervention and control arms) receive a brief educational handout on Parkinson’s disease that is available in the VA’s “Healthwise for Life” handbook [23, 24]. Veterans randomized to the control arm continue to receive care they would have received if they had not enrolled in the study.

### Blinding (masking)

Since the unit of randomization is the patient rather than the physician or clinic, physicians will know which of their patients are receiving the intervention due to information and communication received from the CHAPS NCMs. However, since NCMs foster patient participation in medical care interventions, the risk of bias that can be caused by physician awareness of group assignment is decreased. The intervention is not physician-driven so there is less likelihood of contamination. The advantages of patient-level randomization outweigh any disadvantage [25].

### Data collection

**Fig. 3:**
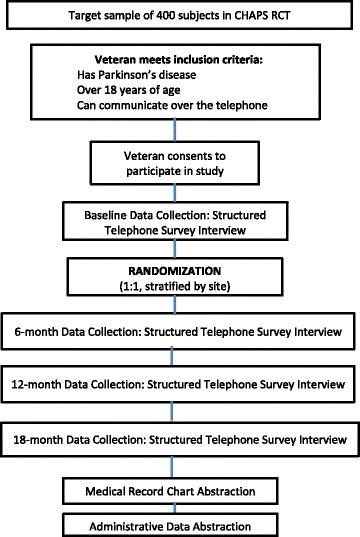
Enrollment of subjects and schedule for collecting evaluation data

Data are obtained by survey interviews with patients and by electronic medical record abstraction. Telephone surveys administered by trained research assistants who are blinded to study arm assignment are conducted at baseline, 6-, 12-, and 18 months (Fig. 3). Study participants receive $25 per survey as recognition for their time. Even though medical record abstractors are not made aware of study arm assignment, these data collectors may become unblinded to randomization status because of the project’s CHAPS name appears in all medical record note titles. However, the standardized chart abstraction form will guard against bias.

Data sources for the primary outcome, adherence to 38 quality indicators, are both medical record abstraction and telephone survey of study participants. The proportion of applicable recommendations followed for each Veteran will be computed and aggregated to the two group levels (intervention and control arms).

Development of guideline adherence measures for those measures not previously operationalized follows a specific process. Each PD indicator or “care goal” is converted to review criteria, which are then operationalized through specific clinical variables and chart review questions [4]. Each observable measurement is mapped to an action/activity performed in the intervention. For example, if the patient is taking antihypertensive medications and has symptomatic orthostatic hypotension, then this medication should be decreased.

### Sample size and level of power

The sample size calculation is based on the primary outcome (Adherence to PD Guidelines Measure using 38 quality indicators, see below and Table 1). Guideline adherence is expressed as the mean across the study participant group of the per-patient percentage of applicable guideline measures for which there was adherence. An applicable PD guideline is one for which a participant is eligible.

**Fig. 4:**
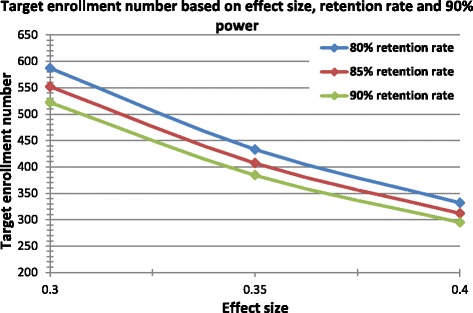
Enrollment sample sizes for achieving analytic sample sizes; range of effect sizes and retention rates

From the literature of chronic disease coordinated care interventions for other conditions, reported effect sizes used for power calculations—and thus reflecting a perceived clinically meaningful difference—range from 0.25 to 0.5 [11, 26, 27]. These are all in the range of a medium effect size per Cohen [28]. Next, the attrition rates of chronic disease coordinated care interventions for other conditions range from 10–20 % [29–32]. Target *enrollment* numbers based on variations of effect size and retention rates of 80–90 % are shown in Table 4 and Fig. 4. Using an alpha of 0.05, 90 % power, and an effect size of 0.35, we calculated an analytic sample size of 346 subjects (173 subjects in each treatment arm). Estimating a retention rate of 85 % corresponds exactly to a target enrollment number of 407. We choose to enroll 400 Veterans to yield an adequate analytic sample size, N = 346, after accounting for the expected attrition rate. Based on the distribution of PD patients across the five VISN 22 VA Medical Centers, we expect that 30.7 % of subjects (n ~ 123) will be enrolled from VA Greater Los Angeles, 23.8 % from VA Loma Linda (n ~ 95), 17.6 % from VA San Diego (n ~ 71), 15.3 % from VA Long Beach (n ~ 61), and 12.5 % from VA Las Vegas (n ~ 50). Enrollees are randomized in a 1:1 ratio to either receive the CHAPS intervention or care as usual.

**Table 4:** Analytic sample sizes, across a range of effect sizes and retention rates

Effect size	Analytic sample size with 90 % power
0.3	2 × 235 = 470
0.35	2 × 173 = 346
0.4	2 × 133 = 266

## Analysis

All analyses will follow the intention-to-treat principle in that participants are included in the analyses regardless of the level of exposure to the intervention activities. In order to accomplish this analysis in patients who drop out of the study, we will employ a conservative imputation method such as last-value-carried-forward. We will consider a p-value less than or equal to 0.05 as statistically significant.

### Primary outcome: quality of care indicators for guideline adherence

The primary outcome is adherence to 38 evidence-based PD quality indicators (Table 1). Guideline adherence will be determined by medical record and interview data. Outcomes are expressed as a percentage of applicable guidelines followed, as identified with quality indicators on chart review. The adherence measure will be treated as continuously scaled.

All multivariate analyses will be preceded by bivariate analyses to describe relationships between key variables. Descriptive statistics will compare the two arms on sociodemographic and clinical characteristics (such as duration of PD) and on study outcome measures at baseline, using two-sample t-tests for continuous variables and χ [2] tests for categorical variables. Baseline characteristics will be compared between participants who complete and participants who do not complete follow-up surveys. Participants who do not finish the final interview due to medical illness or loss to follow-up will be excluded from the analyses but sensitivity analyses will include these participants.

An intention-to-treat analysis on all guideline adherence over time will be conducted using linear regression (or logistic regression when analyzing guidelines individually) with sampling weights using the sandwich variance estimator (robust variance estimator and Huber correction) as implemented in Stata version 11 [33, 34]. Intervention status, indicator variables for site and baseline measures will be included as independent variables in all models. Other potential important covariates will be selected in advance of data analysis to be included in the model.

### Secondary outcome measures

Using the data collected at baseline, 6-, 12- and 18-month follow-up, we will examine the difference in the change score (from baseline to 6 months, baseline to 12 months, and baseline to 18 months) of the secondary outcome measures between two study arms (Fig. 2). These measures will be analyzed using linear regression with sampling weights using the sandwich variance estimator (robust variance estimator and Huber correction) [33, 34]. Intervention status, indicator variables for site and corresponding baseline measures will be included as independent variables in all models, and potential covariates associated with each outcome measure will also be included as independent variables including the adjustment variables as noted above.

Sensitivity analyses will be performed to check the robustness of the main results or to explore other associations. These will include analyses using the last observed outcome measure carried forward to 18 months for those who do not reach the study end point, using only the 6 months of data, using only 12 months of data, and testing whether outcomes change significantly over time within each study arm.

### Process evaluation analyses

A qualitative process evaluation will be conducted to assess implementation process barriers and facilitators to the intervention through review of detailed project meeting minutes from weekly meetings with intervention arm NCM and the clinician neurologist champion at each site. After all evaluation survey data have been collected, the research assistant (RA) will be unblinded to randomization status. The RA will then administer a few qualitative questions to a random sample of intervention arm subjects to get a Veteran-perspective of the quality of care received in the intervention.

### Cost assessment

Using data collected from the Activity logs and itemized start-up costs, we will estimate separately both fixed and variable start-up and implementation costs of the intervention. Total costs for the intervention implementation will be calculated, broken down as technical assistance costs borne by the research team and implementation costs attributable to clinical staff.

### Health services utilization

We will aggregate and compare health services utilization costs between the two study arms. The average cost per patient will be generated based on the administrative data and augmented by selected self-reported healthcare use from the telephone survey. We will calculate the costs associated with health services utilization in the 12 months preceding intervention and during the 18 months of the intervention. Utilization will include physician and emergency room visits, hospital admissions, nursing home admissions, home care services, and pharmacy data. We will determine the units of each service utilized per patient and apply standard unit costs from data costs of VA patient care encounters from the VA Health Economics Resource Center (HERC) [35]. The total cost of each participant will be examined between intervention and usual care using a two-sample *t* test and multivariate regression. Because costs may be skewed, we will also repeat analyses after transforming the dependent variable using square root and logarithmic functions. Because of potential imbalances associated with the high costs of end-of-life care, we will conduct a sensitivity analysis excluding participants who died during the study period.

If the costs for the intervention arm are significantly higher than usual care and significant improvements to patient outcomes are observed, then we may perform a formal cost-effectiveness analysis using the primary and secondary outcomes. In addition, we also have the option of generating quality-adjusted life years (QALYs) based on the Health Utilities Index collected during the telephone interviews.

## Limitations

There is a lack of generalizability inherent in nearly all randomized controlled trials. However, we have few restrictions in inclusion and exclusion criteria, to maximize the applicability of findings to most Veterans with PD, and we are tracking and have some characteristics of those who decline participation.

A challenge may be the complexity of the CHAPS Program itself and the numerous clinical problems that the NCMs, the Veterans, and VA clinicians may need to address in any one patient. However, the CHAPS Program, starting with a thorough structured initial assessment, is designed to proactively identify challenges in order to optimize PD management such as access barriers and the non-motor features of Parkinson’s disease. Identification of these challenges early may decrease preventable complications like medication mismanagement and falls. This leads to simplified clinical care with possibly fewer complications requiring downstream care.

## Protocol modifications

Some modifications have been made to the protocol during the initial months of the study. Early in the implementation phase, we interrupted the study to refine the intervention in response to CHAPS nurse care managers’ feedback.We sought out possible options to better organize the CHAPS assessment and care delivery components. We identified a practical, patient-centered cognitive schema, the Siebens Domain Management Model (SDMM) as a published framework, in clinical use, to assist in the care of individuals with chronic conditions [36–39]. The model, which evolved from earlier work in stroke rehabilitation [40], includes four domains, each with a few subdomains:

I. Medical/Surgical Issues including aspects of disease prevention and organ system function;
II. Mental Status/Emotions/Coping including cognitive function and patient preferences;
III. Physical Function for the full range of patients’ function from basic to intermediate to advanced activities of daily living; and
IV. Living Environment including core elements of the patient’s environment (©Hilary C Siebens MD 2005).

Through consultation with, and permission from, the SDMM creator, we reorganized the CHAPS assessment to follow the SDMM’s 4 domains, as already shown in Table 2. The priority problems, triggered by the assessment, and their corresponding care plans also were ordered using the SDMM (Table 2). Next, the SDMM was integrated into the CHAPS communication templates for Veterans (My Action Plan) and in all the electronic CHAPS medical record MS Word templates for NCM documentation for communicating with physicians and other clinicians. We agreed that this structure would increase the NCM’s ability to identify key health-related issues and make it easy for other clinicians to find relevant CHAPS Program information in the electronic medical record.

In addition, we adopted the 3-ringed binder, the Siebens Health Care Notebook (SHC Notebook) as a better patient self-management tool than a more general binder [41–43]. Four sections, one for each domain, facilitate disaggregating multiple problems into more manageable categories. Also, simplifying the organization of material into four sections can potentially increase Veterans’ self-management ability. Having a piece of information in one section or solving a problem in one area may help solve problems in others. The SHC Notebook is customized for PD by adding one standard PD-related education sheet in each domain section for all patients. NCMs add custom education sheets to individualize these Notebooks depending on patients’ priorities and identified priority problems. Individualized customization also included re-designed “My Action Plans” that incorporated the 4 SDMM-domains.

We will administer 5–10 min surveys to the intervention arm NCMs and clinician champions at each participating VA site about the usability of the Siebens Domain Management Model and the Siebens Health Care Notebook in the intervention. To analyze these data, we will explore common themes guided by the implementation evaluation concepts.

The study has experienced an unexpected decrease in NCM availability due to hiring challenges with bringing identified new nurses on as employees in the VA. Thus, analysis needed to be revised to account for the gap in receipt of the intervention among some of the initial trial cohort. Because of this, a 24-month survey was added to the study for the first 204 enrollees. The remaining subjects (see below for change in sample size) will be followed for the pre-defined 18 months. In order to combine both of these “cohorts” into a complete analysis, a variable will be incorporated into the regression models to reflect the period of time the subject was off the intervention. Re-evaluation of our power analysis indicates a total analytic sample of 266 enrollees (133 in each arm), is required for a medium effect size of about 0.4 SD (Cohen’s terminology) and 90 % power (Table 4). We aim to enroll 320 subjects, estimating attrition around 17 %, to achieve this analytic sample.

## Discussion

The CHAPS Program is a promising approach to proactively address both motor and non-motor health problems in Veterans with Parkinson’s disease. Results from this trial will provide insights on proactive care coordination provided through NCMs for Veterans with Parkinson’s disease. The work has evolved from earlier health services research that identified gaps in care. Basing the program design on the Chronic Care Model facilitated designing a multi-faceted intervention that we anticipate addresses the patients’ clinical complexities effectively. We are helped in this goal by the availability of PD quality indicators and collaboration with experts within and outside the Veterans Affairs to help prioritize these quality indicators.

We also believe that using the SDMM and SHC Notebook will address the care coordination challenge of organizing multiple health-related problems patients may have. This standardized framework and Notebook will facilitate teaching nurses, and others, about Veterans’ care. Second, they may strengthen interdisciplinary communication among patient, family, and health care providers.

The CHAPS Program’s structure, including nurse care managers, standardized assessments and care management protocols may be scalable. Therefore, we will share findings on implementation barriers before the study ends. These may be applicable and relevant to administrators, clinicians, and health services researchers studying other chronic diseases. If the CHAPS Program is demonstrated efficacious, it is poised for dissemination within VA National Parkinson’s Disease Research, Education, and Clinical Center Consortium. Findings will be relevant to all audiences concerned with improving the quality of life of patients with chronic conditions.

## Abbreviations

PD: Parkinson’s disease
CHAPS: Care coordination for health promotion and activities in Parkinson’s disease
VA: Veterans health administration
CCM: Chronic care model
PADRECC: Parkinson’s disease research and education clinical centers
NCM: Nurse care manager
GLA: Greater Los Angeles
VAs: Veterans health administration
APDA: American Parkinson’s Disease Association
LA-CRC: Los Angeles Caregiver Resource Center
NPF: National Parkinson Foundation
PRO: Parkinson’s resource organization
SDMM: Siebens domain management model
SHC: Siebens Health Care
MDS-UPDRS: Movement disorders society—unified Parkinson’s disease rating scale
ADL-UPDRS: Activities of daily living-unified Parkinson’s disease rating scale
WHO: World Health Organization
PD: Parkinson’s disease
PHQ: Patient health questionnaire
ACCESS: Alzheimer’s Disease Coordinated Care for San Diego Seniors
PD-HRQoL: Parkinson’s disease-health-related quality of life
PDQ: Parkinson’s Disease Questionnaire
IADL: Intermediate or instrumental activities of daily living
NMSS: National Multiple Sclerosis Society
MOS: Medical outcomes study
MS: Multiple sclerosis
WHO: World Health Organization
PHQ: Patient Health Questionnaire
UPDRS-ADL: Unified Parkinson’s Disease Rating Scale—Activities of Daily Living
MOS: Medical outcomes study
VISN: Veteran Integrated Service Network
PACIC: Patient Assessment of Care for Chronic Conditions
